# Discriminating Tuberculous Pleural Effusion from Malignant Pleural Effusion Based on Routine Pleural Fluid Biomarkers, Using Mathematical Methods

**Published:** 2017

**Authors:** Reza Darooei, Ghazal Sanadgol, Arman Gh-Nataj, Mehdi Almasnia, Asma Darivishi, Alireza Eslaminejad, Mohammad Reza Raoufy

**Affiliations:** 1 School of Advanced Technologies in Medicine, Isfahan University of Medical Sciences, Isfahan, Iran,; 2 Faculty of Medicine, Shahid Beheshti University of Medical Sciences, Tehran, Iran,; 3 Department of Physiology, Faculty of Medical Sciences, Tarbiat Modares University, Tehran, Iran,; 4 Chronic Respiratory Diseases Research Center (CRDRC), National Research Institute of Tuberculosis and Lung Diseases (NRITLD), Shahid Beheshti University of Medical Sciences, Tehran, Iran,; 5 Faculty of Medicine, Tehran University of Medical Sciences, Tehran, Iran

**Keywords:** Pleural effusion, Malignant pleural exudate, Tuberculous pleural exudate, Weighted Sparse representation-based classification, Decision tree

## Abstract

**Background:**

The differential diagnosis of tuberculous pleural effusion (TPE) and malignant pleural effusion (MPE) is difficult because the biochemical profiles are similar. The present study aimed to differentiate TPE from MPE, using a decision tree and a weighted sparse representation-based classification (WSRC) method, based on the best combination of routine pleural effusion fluid biomarkers.

**Materials and Methods:**

The routine biomarkers of pleural fluid, including differential cell count, lactate dehydrogenase (LDH), protein, glucose and adenosine deaminase (ADA), were measured in 236 patients (100 with TPE and 136 with MPE). A Sequential Forward Selection (SFS) algorithm was employed to obtain the best combination of parameters for the classification of pleural effusions. Moreover, WSRC was compared to the standard sparse representation-based classification (SRC) and the Support Vector Machine (SVM) methods for classification accuracy.

**Results:**

ADA provided the highest diagnostic performance in differentiating TPE from MPE, with 91.91% sensitivity and 74.0% specificity. The best combination of parameters for discriminating TPE from MPE included age, ADA, polynuclear leukocytes and lymphocytes. WSRC outperformed the SRC and SVM methods, with an area under the curve of 0.877, sensitivity of 93.38%, and specificity of 82.0%. The generated flowchart of the decision tree demonstrated 87.2% accuracy for discriminating TPE from MPE.

**Conclusion:**

This study indicates that a decision tree and a WSRC are novel, noninvasive, and inexpensive methods, which can be useful in discriminating between TPE and MPE, based on the combination of routine pleural fluid biomarkers.

## INTRODUCTION

Pleural effusion is a common complication estimated to affect more than 400 people per 100,000 (1). There are two types of pleural effusion, namely transudative and exudative. A transudative pleural effusion develops when the permeability of the capillaries in the lung is altered. Exudative pleural effusion reflects the presence of primary pleural disease and requires etiological investigation (2).

Malignancy and tuberculosis are the leading causes of exudative pleural effusion and account for approximately 50% of all the exudates (3, 4). However, malignant (MPE) and tuberculous pleural effusion (TPE) have similar biochemical profiles and distinguishing between them can be difficult (3, 4). In both types, the pleural fluid is generally lymphocytic, with a predominance of T lymphocytes, particularly CD4-positive T cells (5). Since treatments vary noticeably, a rapid and accurate differential diagnosis is necessary.

Conventional methods, such as thoracentesis and analysis of pleural fluid cytology, histological analysis of tissue obtained via surgical biopsy, image-guided biopsy and local anesthetic thoracoscopy, are not always helpful as they have limitations (2, 6–8). Cytological examinations of pleural fluid can help in diagnosis of 66% of definite cases of malignancy (9). Pleural fluid cultures are positive for mycobacteria in up to 20% of cases and the waiting time for culture results is approximately 1 month (6). Pleural biopsy reveals granulomas in only 46% of cases (9). A combination of the cytological method and biopsy can increase the rate of diagnosis to 73% (9). Even though pleuroscopy could determine the cause of pleural effusion in these patients with 95% accuracy, this facility is invasive and not available in most hospitals (10, 11). Therefore, developing a less-invasive, accessible and early method with high accuracy is greatly needed for diagnosing the causes of pleural effusions.

Previous studies have reported the performance of various biomarkers, such as nucleated cells, lymphocytes, neutrophils, eosinophils, cholesterol, proteins, lactate dehydrogenase (LDH), adenosine deaminase (ADA), interleukin-6 and tumor necrosis factor-α, to differentiate between MPE and TPE (12–14). However, most of these investigations are based on each marker separately, and should be interpreted alongside clinical findings and with the results of other conventional tests (13, 14). It appears that a combination of biological markers can increase the accuracy of diagnosis (12, 13).

Various classification models have been constructed for differentiating between diseases. Sparse representation-based classification (SRC) is a new and powerful data processing method that has shown good performance in the classification of diseases (15–18). In this study, we propose a weighted sparse representation-based classification (WSRC) method, which is a modified version of SRC. WSRC improves the classification accuracy of the system through adding the weights (17).

Making the right decision plays an important role in diagnostic medicine. A decision tree is an effective and reliable supporting tool for decision-making that provides an accurate classification through the use of simple representation of the information gathered. This model consists of starting points (tests or clinical questions) and branches which represent the alternative outcomes of each test or question (19).

The aim of the present study was to differentiate between TPE and MPE using a decision tree and a WSRC method, based on the best combination of routine pleural fluid biomarkers. Moreover, WSRC is compared with the conventional classification methods in terms of classification accuracy.

## MATERIALS AND METHODS

### Data collection

In this research, we undertook a retrospective study of 236 patients with a diagnosis of pleural effusion due to tuberculosis (n=100) or cancer (n=136) who were admitted at Masih-Daneshvari Hospital (Tehran-Iran) between June 2009 and July 2012, after obtaining institutional review board and ethics committee approval.

The cause of pleural effusion was assessed by identifying malignancies in pleural biopsy carcinoma specimens and by identifying granuloma in biopsy specimens, either using positive staining or cultures of mycobacterium tuberculosis with exudate or sputum samples. Additionally, thoracoscopy and video-assisted thoracic surgery (VATS) was undertaken in cases where the diagnosis was unclear.

At the time of admission and before any medical treatment was considered, pleural fluid was analyzed in terms of differential cell count, LDH, protein, glucose and ADA levels. Biochemical measurements were performed using standardized photometric methods (Hitachi models 717,917 or modular DP, Roche Diagnostics Mannheim Germany) and manual microscopy was used for the cell count. Pleural ADA was measured using an automated ultraviolet kinetic test (Roche diagnostic, Barcelona, Spain).

### Sparse Representation-based Classification (SRC)

A SRC classification approach assigns sample vector y as an input, which belongs to an unknown class. This approach is extended to SRC when vector y is being assigned to the class that is represented with training samples and is related to coefficients of sparse representation of y in the most efficient way (15, 20–22).

### Weighted sparse representation-based classification (WSRC)

The discrimination capability of SRC is lost in datasets which distribute in the same direction (18). Distribution of data in the same direction means that the samples with the same vector directions are members of different classes (18). SRC requires normalizing the samples and leads to mapping the samples onto a hypersphere (18). Therefore, data with the same direction distribution are not separable. Although the mentioned normalization is ineffective for the solution of SRC performance, it is an inseparable section of the SRC algorithm. WSRC remedies the limitations of SRC and its performance improves through adding the weights (19). We proposed using the Minkowski distance between the new sample y and the related training samples as weights.

### Support Vector Machine (SVM)

SVM is a conventional supervised learning method that has a favorable performance for classification of high-dimensional data (23). SVM constructs a hyperplane in classifying the data to maximally separate different groups (24). In our analysis, we used the Statistical Pattern Recognition Toolbox for MATLAB.

### Cross-validation

In this study, a leave-one-out cross-validation was performed for evaluating the classification performance of the methods. The function was trained *n* separate times (where *n* is the number of samples) on all the data, except for one sample, in each iteration for which a prediction was made. The average error was calculated to evaluate the performance of methods (25).

### Sequential Forward Selection (SFS)

The Sequential Forward Selection (SFS) method is used to assess the overfitting and to select the best combination of parameters for classification of pleural effusions. First, an empty feature subset is considered. Second, a feature providing the best combination with the already selected features is added in from the rest of the features. This process is continued until all the features are selected (26).

### Decision tree model

A decision tree is a type of supervised learning algorithm that provides a framework for analyzing all possible alternatives for a decision. This model simplifies decision-making in the presence of uncertainty. The tree starts with a node, a main decision, and the lines extend out from this node for each possible solution. If the solution leads to another decision, the new line extends to the next possible series of choices, which provide an overall supportive decision-making process in medicine (19).

### Statistical analysis

We used GraphPad Prism V3.0 (GraphPad Software, San Diego, CA) for the statistical analysis of data. A chi square test, an unpaired t-test, or a Mann-Whitney U-test was used to compare the parameters of groups. Receiver Operating Characteristic (ROC) curves were used to evaluate the power of classification methods for discriminating tuberculous from malignant pleural effusions. P-values less than 0.05 were considered statistically significant.

## RESULTS

The characterizations of patients and pleural fluid biomarkers for each pleural effusion group are shown in Table 1. The proportion of males was similar in the two groups. Patients with MPE were significantly older (p < 0.0001), and had higher RBC count (p < 0.0001), LDH (p = 0.030) and polynuclear leukocyte (p = 0.022) levels in pleural fluid than patients with TPE. In contrast, WBC counts (p = 0.001), lymphocyte (p = 0.001) and ADA (p < 0.0001) levels in pleural fluid were significantly higher in the TPE group compared to the MPE group.

**Table 1.** The characterizations and pleural fluid biomarkers in patients with malignant and tuberculous pleural effusion

	**MPE (n = 136)**	**TPE (n = 100)**	**p value**
**Male, n (%)**	72 (52.94)	65 (65.00)	0.064
**Age, year**	62.21 (14.81)	42.70 (20.70)	< 0.0001
**Red blood cell, ×10^3^/μL**	288.65 (445.89)	105.18 (300.54)	< 0.0001
**White blood cell, ×10^3^/μL**	1.96 (4.60)	4.43 (10.16)	0.001
**Polynuclear leukocytes, %**	22.49 (47.75)	12.83 (22.33)	0.022
**Lymphocyte, %**	77.87 (25.73)	86.31 (22.70)	0.001
**Glucose, mg/dL**	112.85 (67.31)	92.21 (48.05)	0.110
**Protein, mg/dL**	4.34 (1.04)	5.41 (5.12)	0.212
**LDH, IU/L**	726.17 (655.25)	702.61 (479.47)	0.030
**ADA, U/L**	22.21 (15.30)	70.02 (34.63)	< 0.0001

Data are presented as mean (standard deviation)

MPE, malignant pleural effusion

TPE, tuberculous pleural effusion

Table 2 compared the performance of age and various biomarkers of pleural fluid for differentiating TPE from MPE using the WSRC method. ADA yielded the most favorable discriminating ability (sensitivity, 91.91%; and specificity, 74.0%), followed by age (sensitivity, 92.65%; and specificity, 51.0%).

**Table 2.** The performance of age and the biomarkers of pleural fluid in discriminating tuberculous from malignant pleural effusions using WSRC

	Age	RBC	WBC	Poly nuclear	Lymphocyte	Sugar	Protein	LDH	ADA
Se (95% CI)	92.65 (86.89–96.42)	93.38 (87.81–96.93)	98.53 (94.79–99.82)	100.00 (97.32–100.0)	66.91 (58.33–74.74)	69.12 (60.63–76.75)	89.71 (83.33–94.26)	77.94 (70.03–84.59)	91.91 (85.99–95.89)
Sp (95% CI)	51.00 (40.80–61.14)	0.00 (0.00–3.62)	5.00 (1.64–11.28)	0.00 (0.00–3.62)	37.00 (27.56–47.24)	44.00 (34.08–54.28)	17.00 (10.23–25.82)	27.00 (18.61–36.80)	74.00 (64.27–82.26)
LR+ (95% CI)	1.89 (1.54–2.32)	0.93 (0.89–0.98)	1.04 (0.99–1.09)	1.00 (1.00–1.00)	1.06 (0.88–1.29)	1.23 (1.00–1.52)	1.08 (0.97–1.20)	1.07 (0.92–1.24)	3.54 (2.53–4.94)
LR− (95% CI)	0.14 (0.08–0.27)	NaN	0.29 (0.06–1.49)	NaN	0.89 (0.63–1.27)	0.70 (0.50–0.98)	0.61 (0.31–1.17)	0.82 (0.52–1.28)	0.11 (0.06–0.19)
PPV (95% CI)	72.00 (64.73–78.51)	55.95 (49.23–62.51)	58.52 (51.84–64.79)	57.63 (51.05–64.01)	59.09 (50.89–66.94)	62.67 (54.40–70.42)	59.51 (52.45–66.29)	59.22 (51.64–66.49)	82.78 (75.80–88.43)
NPV (95% CI)	83.61 (71.91–91.85)	0.00 (0.00–33.63)	71.43 (29.04–96.33)	NaN	45.12 (34.10–56.51)	51.16 (40.14–62.10)	54.84 (36.03–72.68)	47.37 (33.98–61.03)	87.06 (78.02–93.36)

WSRC, Weighted Sparse Representation based Classification; Se, sensitivity; Sp, specificity; PPV, positive predictive value; NPV, negative predictive value; LR+, positive likelihood ratio; LR−, negative likelihood ratio

**Figure 1.**
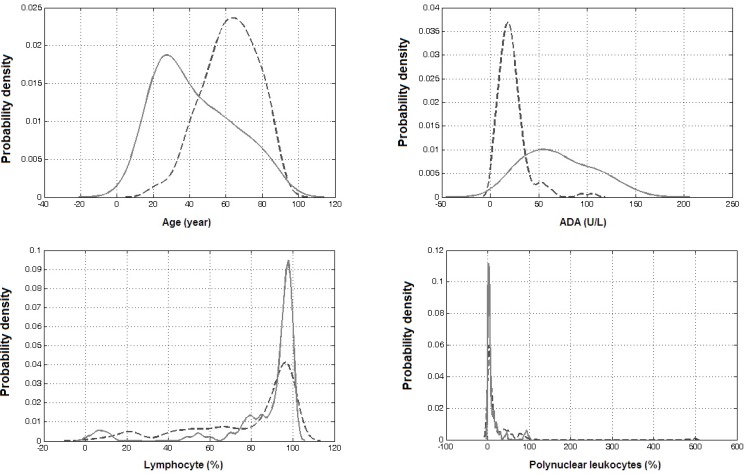
Density estimates of parameters which are used in combination for differentiating tuberculous (continuous line) from malignant (dash line) pleural effusions

The SFS algorithm was employed to obtain the best combination of parameters for the classification of pleural effusion. This optimal set of discriminators not only yields high accuracy with the minimum possible number of parameters, but also offers insight into the factors affecting the classification. The final best combination of parameters for discriminating TPE from MPE included age, ADA, polynuclear leukocytes and lymphocytes. Density estimates of these parameters are shown in Figure 1.

Table 3 shows WSRC, SRC, and SVM performance in differentiating TPE from MPE, based on the combination of four parameters including age, ADA, polynuclear leukocytes, and lymphocytes. The areas under the curves for the all three methods were good. SRC and SVM methods had similar discriminating performance, with the area under the curve of 0.867 (95% CI: 0.816–0.919), sensitivity of 90.44% (95% CI: 84.21–94.81), and specificity of 83.0% (95% CI: 74.18–89.77). However, WSRC outperformed the SRC and SVM methods, with area under the curve of 0.877 (95% CI: 0.826–0.927), sensitivity of 93.38% (95% CI: 87.81–96.93), and specificity of 82.0% (95% CI: 73.05–88.97).

**Table 3.** The performance of classification methods based on the best combination of parameters for discriminating tuberculous from malignant pleural effusions included age, ADA, polynuclear leukocytes and lymphocyte.

	**WSRC**	**SRC**	**SVM**
**Area under curve (95% CI)**	0.877 (0.826–0.927)	0.867 (0.816–0.919)	0.867 (0.816–0.919)
**Sensitivity (95% CI)**	93.38 (87.81–96.93)	90.44 (84.21–94.81)	90.44 (84.21–94.81)
**Specificity (95% CI)**	82.00 (73.05–88.97)	83.00 (74.18–89.77)	83.00 (74.18–89.77)
**Positive Likelihood Ratio (95% CI)**	5.19 (3.41–7.90)	5.32 (3.44–8.23)	5.32 (3.44–8.23)
**Negative Likelihood Ratio (95% CI)**	0.08 (0.04–0.15)	0.12 (0.07–0.19)	0.12 (0.07–0.19)
**Positive Prediction Value (95% CI)**	87.59 (81.09–92.47)	87.86 (81.27–92.76)	87.86 (81.27–92.76)
**Negative Prediction Value (95% CI)**	90.11 (82.05–95.38)	86.46 (77.96–92.59)	86.46 (77.96–92.59)

SRC, Sparse Representation based Classification

WSRC, Weighted Sparse Representation based Classification

SVM, Support Vector Machine

**Figure 2.**
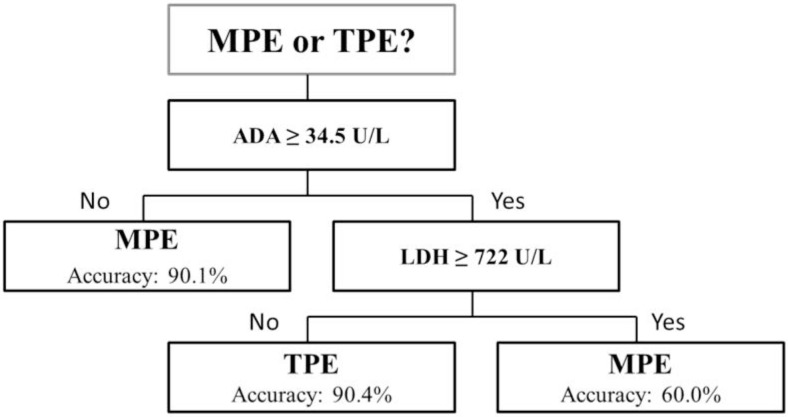
The generated flowchart of decision tree to discriminate the tuberculous from malignant pleural effusions. The total train and test accuracy of generated decision tree were 88.8% and 87.2%, respectively. MPE, malignant pleural effusion; TPE, tuberculous pleural effusion

Figure 2 displays the suggested decision tree to discriminate between the tuberculous and the malignant effusions. The generated flowchart of the decision tree had a train accuracy of 88.8% and test accuracy of 87.2%.

## DISCUSSION

Assessing biomarker levels in pleural fluid is an alternative noninvasive approach in differentiating between the different causes of pleural exudate. In the present study, the routine biomarkers of pleural fluid were measured in patients with malignant and tuberculous pleural effusions. ADA provided the highest diagnostic performance in differentiating TPE from MPE. However, we found that the discriminant ability improved when parameter combinations, including age, ADA, polynuclear leukocytes and lymphocytes, were applied. Moreover, WSRC had a better performance in classifying pleural effusion compared to the SRC and SVM methods. Also, we developed a simple and accurate decision model for differentiating between tuberculosis and malignant pleural effusion.

Thoracentesis, with pleural fluid examination, is the first step in the work-up of every pleural effusion of unknown origin. Total and differential cell counts, and biochemical studies (including total proteins, LDH, glucose, and ADA), are routinely conducted on the pleural fluid samples in hospitals (11). Our results showed that, although both malignant and tuberculous pleural fluids were lymphocytic, a higher proportion of lymphocytes was observed in the tuberculosis group compared to the malignant group. Also, the percentage of polynuclear leukocytes in MPE was significantly higher than in the TPE group. In addition, patients with MPE were older and had elevated LDH and hemorrhagic fluid levels than those in the TPE group. In contrast, ADA in TPE was significantly higher than in MPE. Despite similar findings in other previous reports (12, 27), these parameters do not permit differentiation between MPE and TPE because of overlapping values. For instance, an extremely high ADA activity is highly suggestive of lymphoma rather than TPE. Therefore, lymphomatous pleural effusion may be more difficult to differentiate from TPE in patients with a negative pleural fluid cytological examination result (27–29).

However, numerous studies have shown that ADA of pleural fluid, an enzyme produced by macrophages and activated T lymphocytes (28), is a valuable biochemical marker, which has a high sensitivity (87 to 100%) and specificity (81 to 97%) for the diagnosis of TPE (29–36). In agreement with previously mentioned studies, we found that ADA discriminated well between TPE and MPE, with 91.91% sensitivity and 74.0% specificity. Despite the high sensitivity of ADA, its diagnostic specificity is influenced through the local prevalence of tuberculosis, laboratory methodology, population ethnicity and other clinical conditions (37, 38).

Two previous studies applied a combination of parameters for discriminating between different causes of exudative pleural effusion (12, 13). Daniil et al (13) measured ADA, interferon-γ, C-reactive protein (CRP), carcinoembryonic antigen (CEA), interleukin-6, tumor necrosis factor-α and vascular endothelial growth factor (VEGF) levels in pleural fluid from patients with exudative pleural effusion. They used a multinomial logit model and found that the combination of ADA and CRP levels might be sufficient for establishing a diagnosis of exudative pleural effusion, whereas inclusion of interferon-γ could be an alternative option. However, their results were inconclusive, because the number of cases was relatively low in comparison with the number of parameters used for discrimination. Valdés et al (12) recently discriminated between different causes of exudative pleural effusion with a high diagnostic accuracy using a combination of age, tumor necrosis factor-α, LDH, ADA, CRP and CEA. They developed a polytomous model that could classify a high proportion of patients with TPE (85.8% sensitivity and 94.4% specificity) and MPE (81.6% sensitivity and 87.3% specificity). Both these studies used biomarkers that are not routinely evaluated in pleural fluid samples in hospitals. In the present study, we measured ADA of pleural fluid as well as routine biomarkers which assess every pleural effusion of unknown origin in the work-up. The SFS algorithm was then used to select the best combination of parameters for classifying pleural effusion. The WSRC method achieved a good diagnostic performance in differentiating TPE from MPE (93.38% sensitivity and 82.0% specificity) when a parameter combination, including age, ADA, polynuclear leukocytes and lymphocytes, was applied.

Advances in mathematical learning methods have led to the development of some high-dimensional classification algorithms which have been recently used in the medical sciences. The improvements in these technologies could help enhance disease identification accuracy. As a result, various classification models have been constructed for differentiating between diseases. Among these, supervised machine-learning techniques, in which a training procedure is used to create a classification model for testing, are the most-widely used (39, 40). SVM is a conventional supervised learning method that has a favorable performance for classification of high-dimensional data (41). However, it has a limitation in dealing with noisy data and, as with other supervised learning methods, is a requirement on many labeled training samples (41). On the other hand, to improve classification robustness in respect of noises, a sparse representation technique has been proposed and has been successfully applied to various classification problems (15–17). The principal addition of SRC is to represent a new sample using the least number of training samples (15). Since SRC does not contain separate training and testing stages, as in the supervised learning method, this method has no overfitting problem (17). However, the discrimination capability of SRC is lost in datasets that are distributed in the same direction (18). In this study, the SRC prototype classification method has been modified through adding the weights (WSRC) for solving some of the dataset problems and improving the classification accuracy of the system (19). As expected in theory, our experimental results showed that adding weights can enhance the performance of the SRC method. According to our results, WSRC outperformed the SRC and SVM methods in classification of pleural effusions.

A decision tree is a reliable and effective decision-making model which provides an accurate and simple representation of gathered knowledge. This model can easily be validated during the decision-making process by an expert. Therefore, decision trees are applicable in decision-making processes in medicine. In this study, the decision tree was only used for the differential diagnosis of TPE and MPE, and diagnosis of other sources of pleural effusion are based on clinical evaluations.

Some limitations of this study should be acknowledged. First, we differentiated only TPE and MPE patients. Although most problems occur in distinguishing between exudative effusion in these two diseases (3, 4), future investigations should consider including all causes of pleural exudate. Second, the usefulness of WSRC in differentiating TPE from MPE was not tested in real time. It is not clear how physicians would respond if WSRC could distinguish the cause of pleural effusion. Third, the present study was carried out at a single medical center. These findings must be corroborated on patients from multiple locations, using more samples.

In conclusion, a decision tree and a WSRC are both novel, noninvasive, and inexpensive methods, which can provide highly effective and reliable structures useful for discrimination between TPE and MPE, based on a combination of routine pleural fluid biomarkers. The present study indicates that these applied mathematical methods can provide high diagnostic success rates to assist in the diagnoses of exudative pleural effusion in patients waiting for laboratory outcomes of pleural tissue, and for treatment planning.
